# Hip-Spine Syndrome in Patients With Spinal Cord Injuries: Hyperlordosis Associated With Severe Hip Flexion Contracture

**DOI:** 10.3389/fped.2021.646107

**Published:** 2021-07-12

**Authors:** Isaac Rhee, Woo Sung Do, Kun-Bo Park, Byoung Kyu Park, Hyun Woo Kim

**Affiliations:** ^1^Melbourne Medical School, University of Melbourne, Melbourne, VIC, Australia; ^2^Division of Pediatric Orthopedic Surgery, Severance Children's Hospital, Yonsei University College of Medicine, Seoul, South Korea; ^3^Department of Orthopedic Surgery, Haeundae Paik Hospital, Inje University College of Medicine, Busan, South Korea

**Keywords:** spinal cord injury, paraplegic, hip flexion contracture, lumber hyperlordosis, hip-spine syndrome

## Abstract

**Aim:** Spinal cord injury (SCI)-related flaccid paralysis may result in a debilitating hyperlordosis associated with a progressive hip flexion contracture. The aim of this study was to evaluate the correction of hip flexion contractures and lumbar hyperlordosis in paraplegic patients that had a history of spinal cord injuries.

**Methods:** A retrospective review was performed on 29 hips of 15 consecutive patients who underwent corrective surgeries for severe hip flexion deformity from 2006 to 2018. The mean age at surgery was 10.1 years (2.7 to 15.8), and the mean follow-up was 68 months (7 to 143). Relevant medical, surgical, and postoperative information was collected from the medical records and radiographs.

**Results:** Improvements were seen in the mean hip flexion contracture (*p* < 0.001) with 100% hip correction at surgery and 92.1% at the latest follow-up. Mean lumbar lordosis decreased (*p* = 0.029) while the mean Cobb angle increased (*p* = 0.001) at the latest follow up. Functional score subdomains of the Spinal Cord Independence Measure, Functional Independence Measure, and modified Barthel activities of daily living (ADL) scores remained the same at the final follow-up.

**Conclusion:** For paraplegic SCI patients, we found an association between treating the hip flexion contracture and indirect correction of their lumbar hyperlordosis. We recommend the surgeon carefully examine the hip pathology when managing SCI-related spinal deformities, especially increased lumbar lordosis.

## Introduction

Flaccid paralysis after spinal cord injuries (SCIs) may result in variable degrees of loss of function and progressive deformities of the spine and lower extremities. Inhibited muscular actions and resultant muscle shortening cause myostatic contractures, which, along with neuromuscular imbalances, gravitational stresses, and prolonged shortened malposture, produce joint deformities (1). Especially when SCIs occur in the growing child, the challenges that arise are more problematic and differ from those experienced in adulthood; a culmination of abnormal stress and strain exerted and the rapid changes in the architecture of the bones and joints debilitate the child, and a vicious cycle ensues (1–4).

SCI-related flaccid paralysis mimics the clinical features of poliomyelitis, and an iliotibial band (ITB) contracture is seen in both (5, 6). Yount (7) and Irwin (8) reported that the poliomyelitis-related ITB contracture is the greatest deforming factor in the lower extremity, resulting in an intractable sequela of the hip. The ITB occupies a plane lateral and anterior to that of the hip joint, and as such, a flexion and abduction hip deformity occurs, and for comfort, the hip is externally rotated. Additionally, in a growing child, the discrepancy between the increasing length of the lower extremity and contracted ITB causes a progressive flexion and valgus deformity at the knee (4, 7, 8).

The bilateral hip flexion contractures in SCI may also anteriorly angulate the pelvis and cause a compensatory increase in lumbar lordosis (LL) to maintain the trunk upright while sitting (2, 6, 8–10). However, these deformities eventually render transferring, positioning, and sitting tolerance in a wheelchair to be difficult, and complications of skin hygiene, pressure sores, and poor self-esteem arise (5, 11, 12). In the supine position, the forces of gravity acting on the flail lower limbs necessitate an awkward frog leg attitude when lying and result in great discomfort and the requirement of support under their legs when asleep. Unfortunately, stretching and manipulation rarely provide effective relief for this hip deformity in the growing child (2, 4, 13).

To date, there have been no reports that exclusively manage the paraplegic SCI-associated hip deformity and exaggerated LL. Few have reported operative results of spinal deformities in SCI and poliomyelitis, of which most focus on scoliosis (14). Furthermore, despite the relationship between the hip and spine being briefly mentioned in the literature, the secondary effects of treating the spine by hip deformity correction have not been investigated. The purpose of this study was to review our experiences with the treatment of ITB contracture-related severe hip joint contracture and associated hyperlordosis in flaccid patients with SCI.

## Materials and Methods

This retrospective study was approved by our Institutional Review Board. Between April 2006 and December 2018, 15 consecutive patients with wheelchair-bound SCI-associated paraplegia and a diagnosis of severe flexion, abduction, and external rotation contractures of the hip and lumbar hyperlordosis underwent sequential hip deformity correction surgery. We reviewed their medical records and plain radiographs of the spine and lower extremities. The etiology of SCI, the level of spinal cord lesion, and the lists of surgical managements performed including past, index, and subsequent operations were recorded.

**Figure 1:**
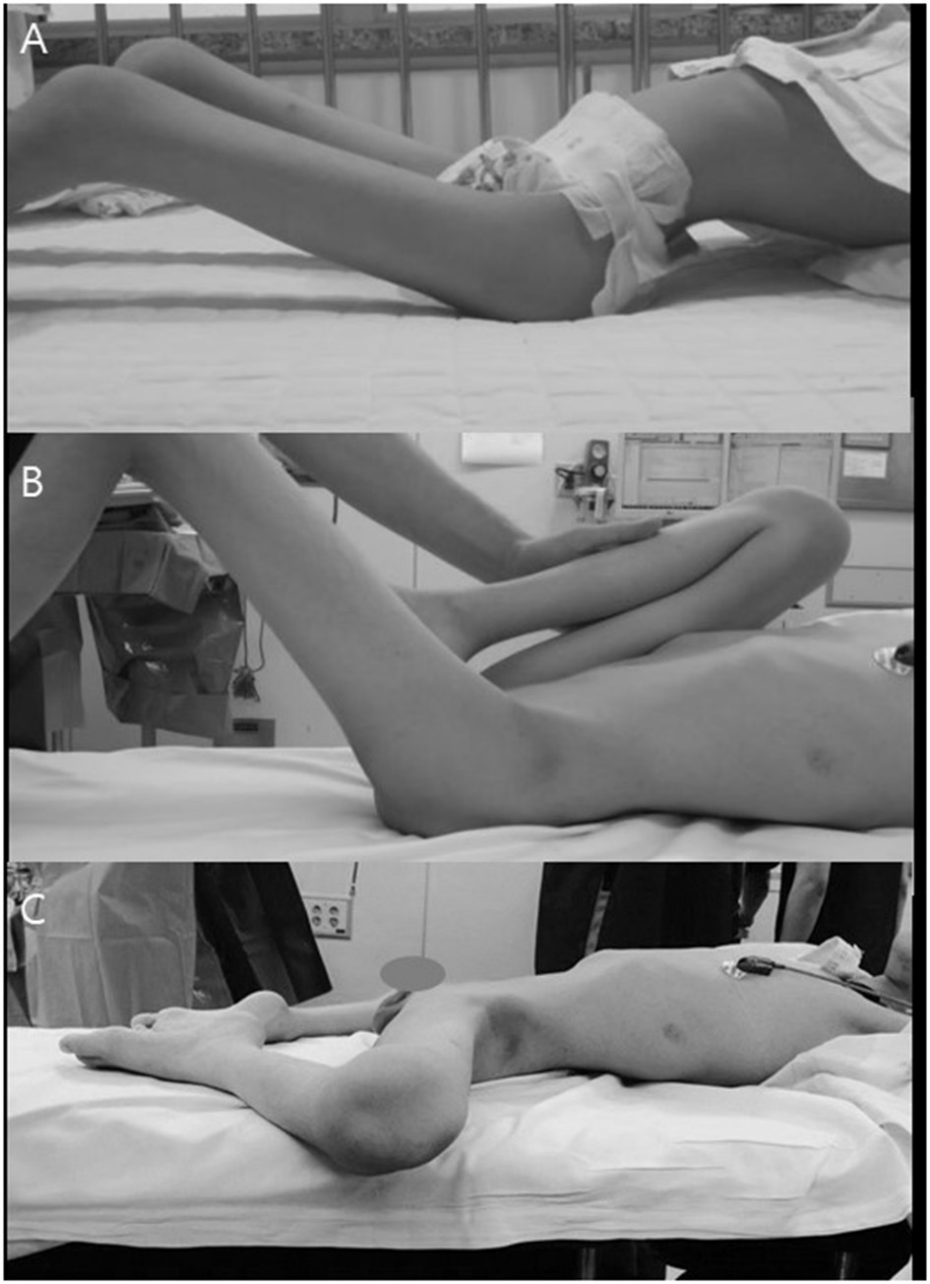
Patient with hip flexion–abduction–external rotation contracture. **(A)** Compensatory increased LL due to their hip contracture. **(B)** Thomas test to examine the degrees of real hip flexion contracture. **(C)** Frog leg attitude when lying to improve comfort and reduce the increased lordosis.

The surgical indication was a hip that failed to be treated with conservative physiotherapy and led to difficulties with transferring, upright positioning, lying, and sitting tolerance for the patient. Additionally, when lying in bed, all the patients excessively abducted their hips to reduce the hyperlordosis and provide more comfort (Figure 1). All operations were performed by a single surgeon (H.W.K.).

The choice of surgical procedure was based on the severity of the hip deformity and the degrees of correction achieved after each step of the operation. Initially, the Thomas test (Figure 1) (15) was performed to appreciate the degrees of hip flexion contracture of each hip; the examined hip was neutrally rotated, and the contralateral neutrally rotated hip was gradually flexed until the exaggerated LL had diminished. We then proceeded to sequentially release all the contracted soft tissues around the hip joint as follows.

An oblique incision, for the traditional anterolateral approach, to the hip was made medial and distal to the anterior superior iliac spine and extended laterally above the greater trochanter. The sartorius was detached from its origin of the anterior superior iliac spine, the rectus femoris was detached from its origin of the anterior inferior iliac spine, and the proximal tensor fasciae lata was divided from its anterior border posteriorly. An additional lateral longitudinal incision, proximal to the femoral condyle, was made to expose the distal tensor fascia lata. The distal ITB and fascia lata were divided posteriorly to the biceps tendon and anteriorly to the midline of the thigh, and a segment of the tight ITB was excised.

**Figure 2:**
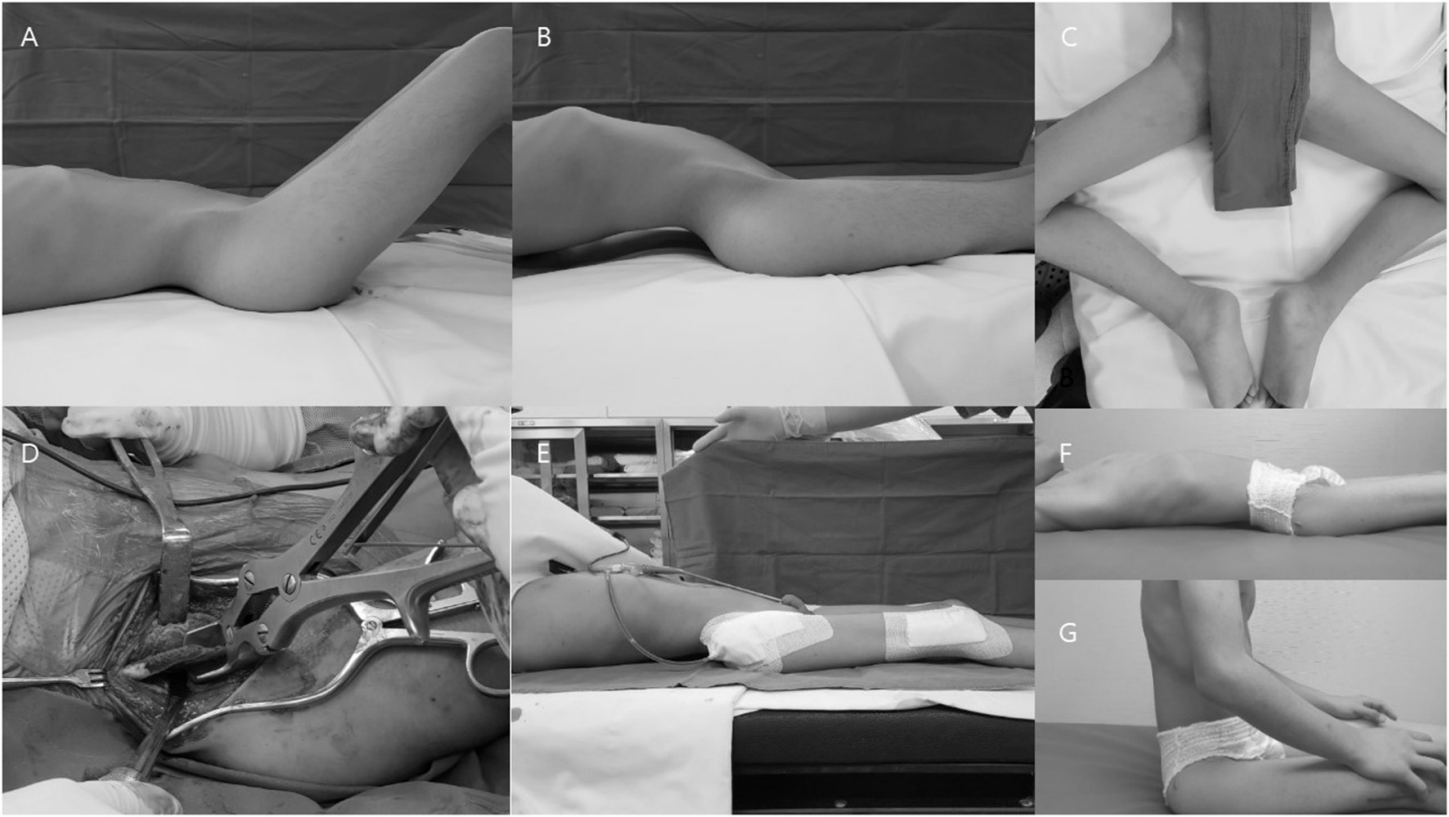
Representative images of a patient who underwent proximal and distal extensive soft tissue release with an additional iliac crest ostectomy. **(A)** Hip flexion contracture. **(B)** Compensatory hyperlordosis. **(C)** Abduction and external rotation of the hip for comfort when lying. **(D)** Additional iliac crest ostectomy. **(E)** Disappearance of the lumbar hyperlordosis after surgery. **(F,G)** Patient experienced improved comfort when lying and sitting.

Psoas tendon was then recessed over the pelvic brim, and the muscle mass anterior to the hip joint capsule was completely released and debulked. An additional anterior hip capsulotomy was performed in selected patients in order to facilitate further hip extension, and the gluteus medius and minimus and short external rotators were detached from their trochanteric insertions in all patients. The hip was then examined again to evaluate the degrees of correction achieved, and any residual soft tissue contractures were further released. In severe cases with inadequate correction by the above-mentioned procedures, an additional iliac crest ostectomy was further completed using the abductor slide technique (Figure 2) (16).

**Figure 3:**
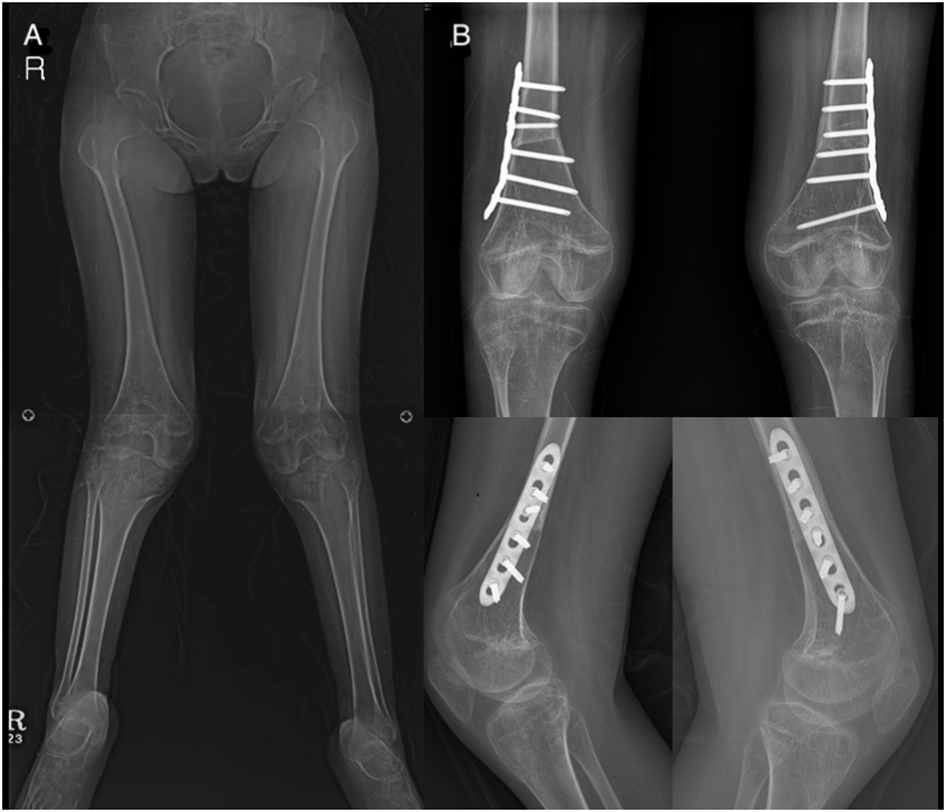
Patient (no. 14) who underwent concomitant knee surgeries. **(A)** Genu valgum deformity. **(B)** Correction with femoral extension and varus osteotomy.

For patients and parents that desired concomitant knee correction for comfortable sitting in a wheelchair, we further corrected the valgus and/or flexion deformity of the knee, acutely with a femoral extension/shortening and varus osteotomy (Figure 3) (17, 18) or gradually by a temporary hemiepiphysiodesis technique (18, 19). Postoperatively, the patients were immobilized with an above-knee hip spica cast for 3 weeks, with the hip flexed about 20° to lessen the tension exerted on the incisional wound. After the removal of the cast, a comprehensive rehabilitation program was initiated.

Physical examinations were performed each in the natural recumbent position of the hip and with forceful extension of the hip joint in order to appreciate the amount of real hip flexion contracture and LL. Preoperative, immediate postoperative, and final follow-up plain radiographs and clinical photographs were also reviewed. Standard radiographic measurements of LL (L1-S1) (20), Cobb angles (21, 22), and the status of the hip joint with regard to the existence of hip subluxation or dislocation were also recorded using anteroposterior pelvic and spinal radiographs and long spinal column lateral radiographs.

We reviewed the functional outcome instruments of the Spinal Cord Independence Measure (SCIM), Functional Independence Measure (FIM), and modified Barthel Index, which were measured before and after surgery by the physiotherapists at our tertiary rehabilitation center (23, 24). In this study, we specifically used subdomains of each functional measure related to mobility, wheelchair, and toileting.

Data were presented as mean ± SD (range). Paired *t*-test and Wilcoxon signed rank test were used, and a *p*-value of <0.05 was defined as statistically significant.

## Results

There were six males and nine females, and the mean age at the time of the index operation was 10.1 years (2.5 to 15.7). The mean follow-up period was 68 months (7 to 143), and the mean age at the latest follow-up was 15.6 years (7.5 to 22.3). All but one patient underwent bilateral surgeries, and the details of the patients are presented in Table 1.

**Table 1:** Information of patients.

**Patient number/sex**	**Age at surgery (Y + M)**	**Age at F/U (Y + M)**	**Etiology of SCI**	**Last intact level (R/L)**	**Operative procedures**	**Hip flexion contracture (R/L**, ^****°****^**)**		**Lumbar lordosis (**^****°****^**)**		**Cobb angle (**^****°****^**)**		**Functional score (SCIM/FIM/MBarthel)**						**Comments**
						**Pre**	**Final F/U**	**Pre**	**Final F/U**	**Pre**	**Final F/U**	**Pre**			**Final F/U**			
1/F	2 + 7	13 + 5	Neuroblastoma	L3/T12	(L)S	0/45	0/0	64.9	38	32.1(L1-L5)	51.7(T11-L5)	15	22	20	15	23	20	Left hip dislocated at final F/U
2/F	5 + 1	12 + 2	Guillain-Barre syndrome	T12/L1	(B)S + C + (R)DFE	40/40	0/0	29.4	48	0	49.3(T9-L3)							Heterotopic ossification
3/M	6 + 2	7 + 6	Transverse myelitis	T7/T7	(B)S + C + DFE + (R)IO	50/50	0/0	58.2	38.8	8.5(T11-L5)	15.4(T11-L5)	16	18	17	16	18	17	Left hip subluxated at final F/U Heterotopic ossification
4/M	6 + 9	11 + 5	Ependymoma	T11/T11	(B)S + (R)DFE + (L)PTE	45/45	0/0	48.8	53.6	7.5(L3-L5)	51.7(T12-L5)	16	23	19	16	23	19	
5/F	7 + 8	16 + 10	Trauma	T10/T10	(B)S + (L)DFE	50/50	0/0	27.6	24.5	13.9(T7-L5)	35.9(T5-T12)	13	18	16	13	18	16	
6/M	9 + 2	15 + 2	Trauma	T9/T10	(B)S + C + DFE	40/40	0/0	80.8	64.2	33.5(T8-L3)	41.9(T5-T11)	18	26	20	18	26	20	
7/F	9 + 2	20 + 1	Transverse myelitis	T12/T10	(B)S + (R)C	50/50	0/20	49.8	43.9	9.4(L2-L5)	29.3(T11-L4	17	23	20	17	23	20	Right hip dislocated at final F/U
8/M	10 + 3	10 + 10	Trauma	T12/T12	(B)S + C	35/35	0/0	79.1	42	25.6(T12-L4)	10.8(T12-L4)							
9/F	10 + 4	22 + 3	Transverse myelitis	T7/T7	(B)S + DFEO + TDO	55/55	0/0	31.7	22.5	28.8(T11-L5)	71.4(T11-L5)	17	26	20	17	26	20	Scoliosis surgery 4 years after index op
10/F	10 + 10	15 + 10	Transverse myelitis	T8/T10	(B)S	40/40	0/0	47.4	29.1	25.2(T7-L5)	43.1(T7-L5)							
11/F	13 + 1	21 + 8	Transverse myelitis	T10/T10	(B)S + C	35/35	0/0	66.4	34.6	49.2(T11-L5)	85.5(T12-L5)	17	21	20	17	23	20	Scoliosis surgery 1 + 3 years after index op
12/M	13 + 6	15 + 11	Transverse myelitis	C4/C4	(B)S + (R)C	45/45	0/0	34.2	18.2	81(T9-L5)	110.0(T9-L4)	16	23	20	16	23	20	
13/M	14 + 7	16 + 0	Subdural hemorrhage	T9/T9	(B)S + C + IO	55/55	20/20	76.4	35.3	44.4(T11-L5)	65.1(T11-L5)	15	18	13	15	18	13	
14/F	14 + 7	15 + 6	Teratoma	L2/L2	(B)S + IO + DFEVO	40/40	0/0	81.6	67.7	21.2(T11-L4)	18(T8-T12)	13	16	17	13	16	17	
15/F	15 + 7	19 + 8	Unknown	Unknown	(B)S	60/60	35/35	78.8	83.8	54.4(T6-L5)	48.3(T6-L5)	17	26	20	17	26	20	Scoliosis surgery prior to index op

*M, male; F, female; Y, years; M, months; F/U, follow-up; SCI, spinal cord injury; R, right; L, left; Pre, preoperative; SCIM, spinal cord independence measure; FIM, functional independence measure; MBarthel, modified barthel ADL index; S, extensive soft tissue release; C, additional capsulotomy; IO, iliac wing ostectomy; DFE, distal femur epiphysiodesis; PTE, proximal tibia epiphysiodesis; DFEO, distal femoral extension osteotomy; TDO, tibial derotational osteotomy; DFEVO, distal femoral extension/varus osteotomy; T, thoracic; L, lumbar*.

In 14 hips of eight patients, an anterior hip capsulotomy was completed, and an additional iliac crest ostectomy was performed in five hips of three patients for the correction of residual hip flexion-abduction contractures despite aggressive soft tissue releases. Concomitant osseous procedures performed included distal femoral extension/varus osteotomy for valgus and/or flexion deformities at the knee in four femurs of two patients, and temporary hemiepiphysiodesis for gradual correction of genu valgum in seven femurs of five patients, due to their open growth plates at the time of surgery.

**Figure 4:**
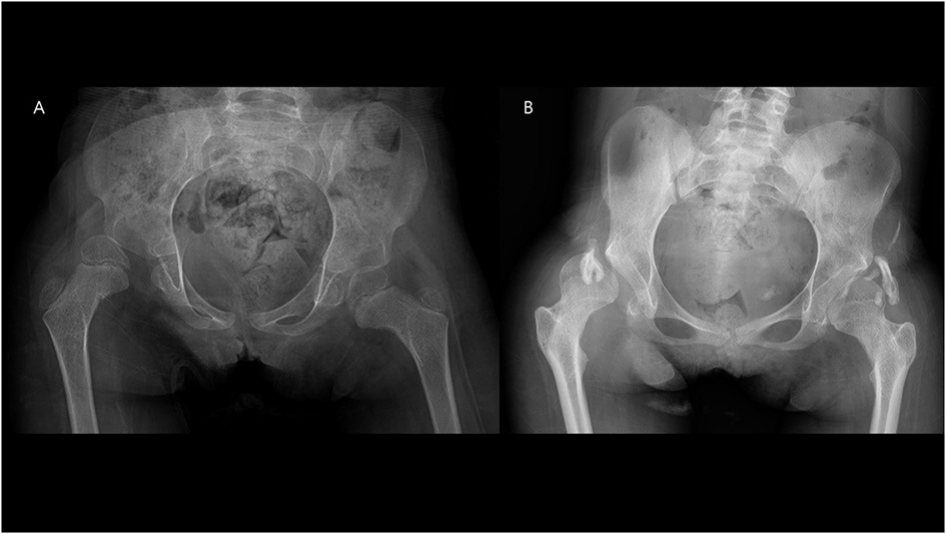
**(A,B)** Patient (no. 2) with heterotopic ossification noted at the final follow-up.

The mean hip flexion contracture improved significantly from 45.7° ± 7.4° (35 to 60) preoperatively to 4.5° ± 10.2° (0 to 35) at the final follow-up (*p* < 0.001). The percentage of hip flexion contracture correction was 100% at the time of surgery and found to be 92.1% at the final follow-up. Before surgery, all patients had at least a hip subluxation or dislocation in one side: 22 subluxations, one dislocation, and seven hips without hip displacement. At the time of the final follow-up, the degree of hip displacement was not changed in 27 hips. However, two subluxated hips of two patients before the index operation progressed into dislocations and one non-subluxated hip into a subluxation; this patient was the youngest of the cohort operated at the age of 2 years 7 months and experienced unilateral subluxation. One patient had previous spinal fusion before the index operation at another hospital due to disabling hyperlordosis; however, the clinical problems induced by the severe hip flexion contracture remained despite a trial of fusion. Postoperative heterotopic ossification around the hip joint was noted in three hips of two patients (Figure 4).

**Figure 5:**
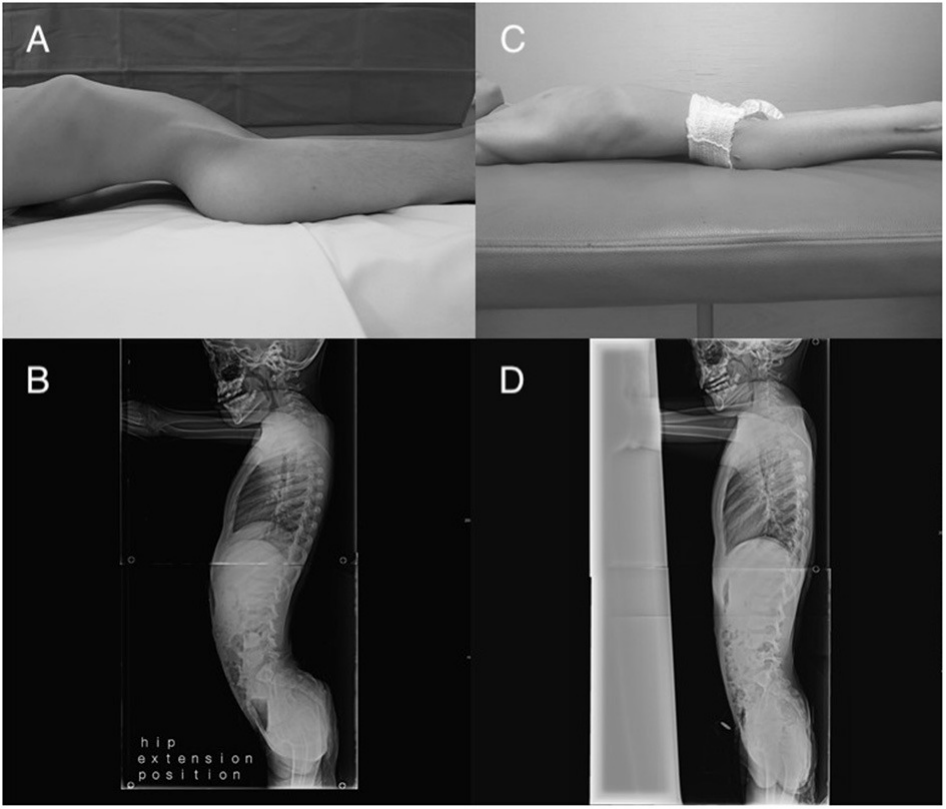
Patient (no. 3) with **(A,B)** lumbar hyperlordosis observed when lying before surgery. Patient experienced **(C)** improved comfort and **(D)** disappearance of hyperlordosis after surgery.

**Figure 6:**
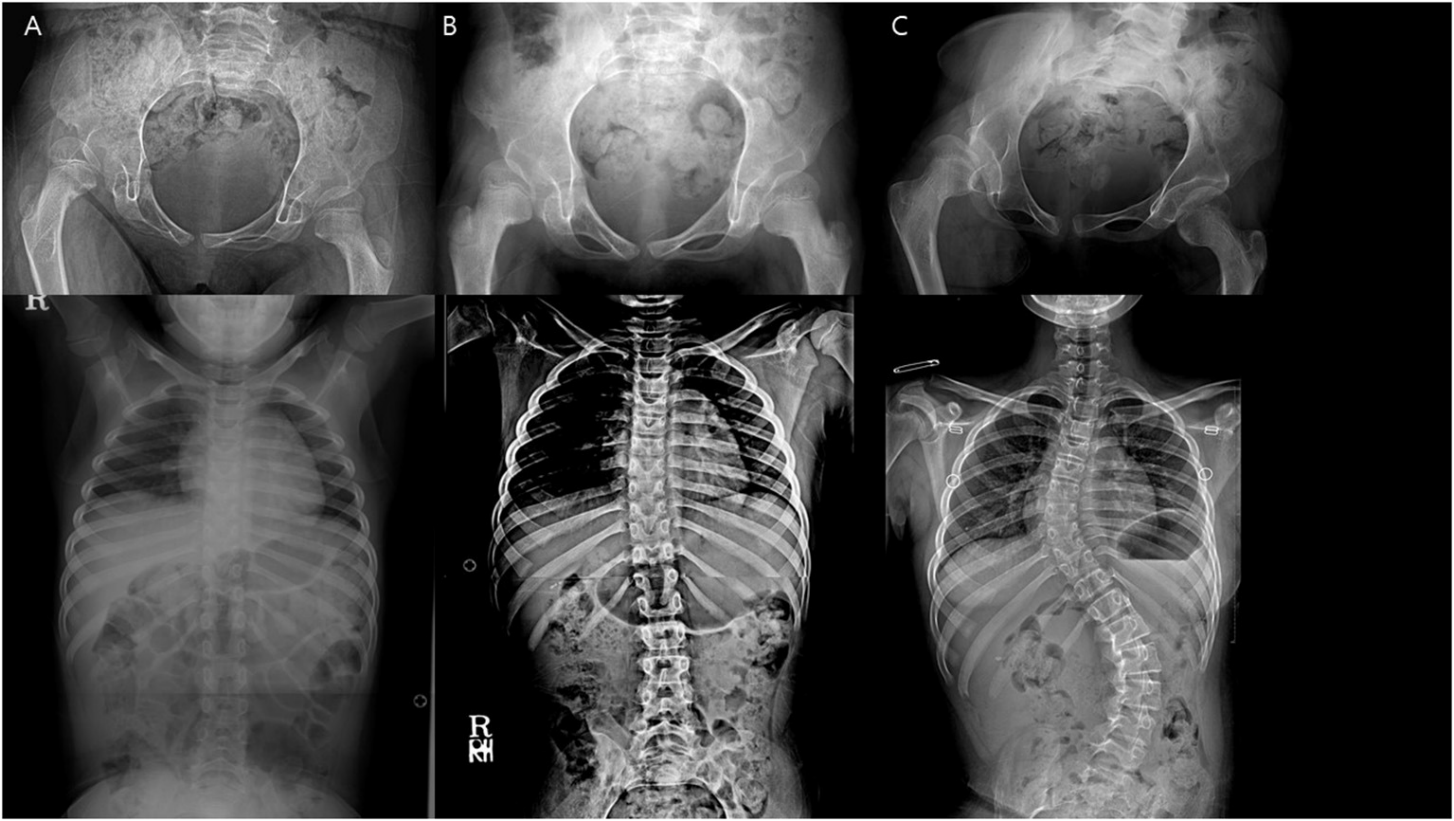
A patient with newly developed scoliosis after surgery and stable hip status throughout. **(A)** Preoperative. **(B)** 1 year after the index operation. **(C)** 5 years after the index operation.

**Figure 7:**
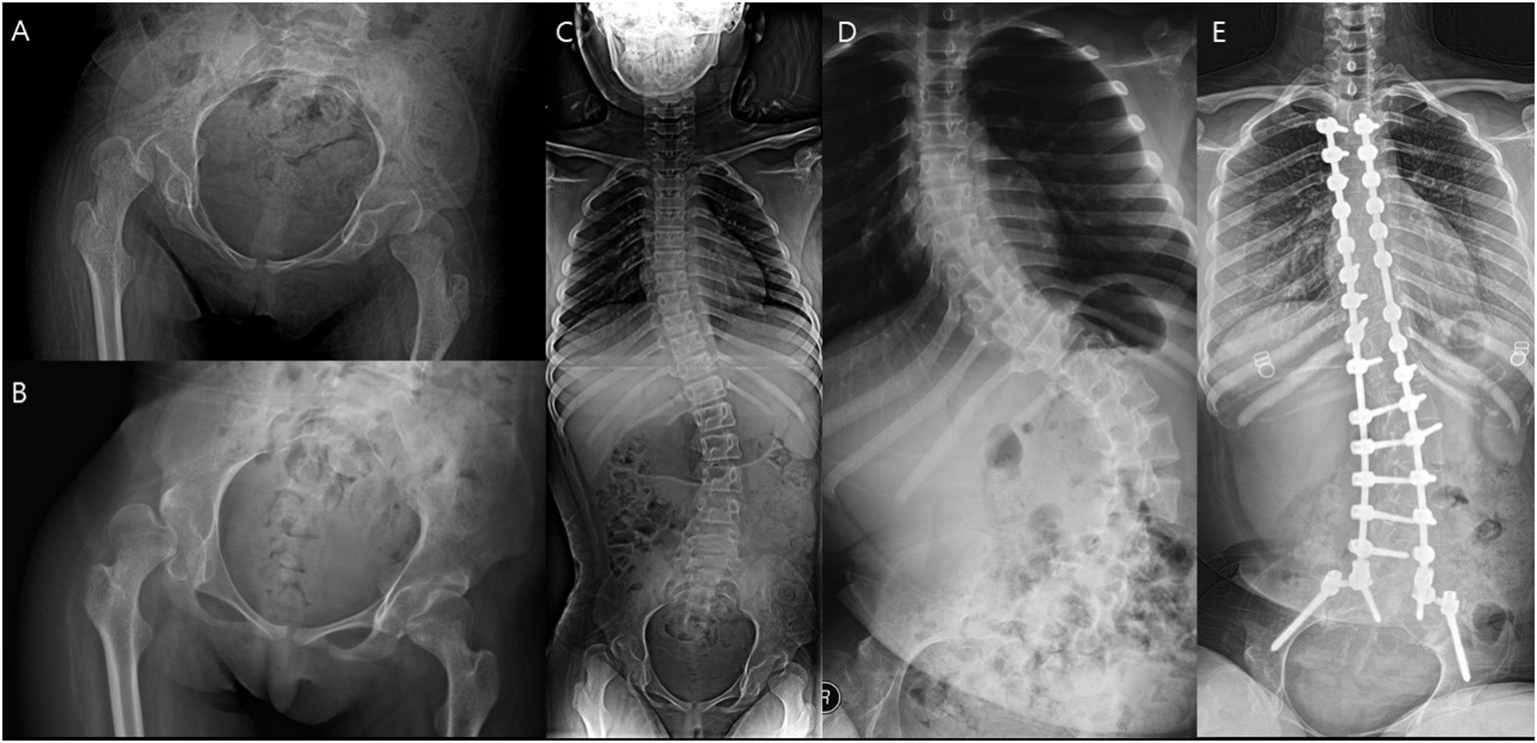
A patient who underwent spinal fusion after the index surgery. **(A)** Preoperative hip. **(B)** Hip 1 year after surgery. **(C)** Preoperative spine. **(D)** Aggravated scoliosis 1 year after surgery. **(E)** Spinal fusion performed 4 years after the index operation.

The mean LL improved significantly from 57.0° ± 19.4° (27.6 to 81.6) before surgery to 42.9° ± 17.5° (18.2 to 67.7) at the final follow-up (Figure 5), with a mean 21.3% decrease (*p* = 0.029). Preoperatively, mean Cobb angle was 28.4° ± 20.1° (0 to 81), and the final Cobb angle was 48.5° ± 26.8° (10.8 to 110) (*p* = 0.001). All but three patients were found to have increased Cobb angles at the final follow-up. Four patients had a Cobb angle less than 10° before surgery, and all of them had newly developed scoliosis without hip displacements at the final follow-up (Figure 6). Two patients required spinal deformity correction after the index operation due to their progressive aggravating scoliosis (Figure 7), from 28.8° before surgery to 71.4° at the time of spinal fusion, and from 49.2° to 85.5°, respectively.

The mean SCIM, FIM, and modified Barthel Index subdomain scores were not changed after surgery: from 15.8 ± 1.5 (13 to 18), 21.7 ± 3.3 (16 to 26), and 18.5 ± 2.2 (13 to 20) preoperatively to 15.8 ± 1.5 (13 to 18), 21.9 ± 3.4 (16 to 26), and 18.5 ± 2.2 (13 to 20) at the final follow-up (*p* = 1.000, *p* = 0.18, and *p* = 1.000, respectively).

## Discussion

The current study describes the experiences of a single institution with treating SCI-associated hip flexion deformity over a 13-year period. To date, there have been no reports investigating the efficacy of the surgical correction of such deformities. In our experience, severe hip contractures lead to an increased LL, and the treatment of the primary hip deformity subsequently corrects the associated sagittal plane deformity of the spine.

All the hip flexion deformities of the patients were completely corrected by a sequential step-by-step procedure, and the degrees of correction were noted to be 92.1% at the latest follow-up. Residual hip flexion contractures were seen in three patients, and this may be attributed to the expected physiological influences of the rapidly growing skeleton, as these patients were young at the time of surgery. Flaccid paralysis with SCI or poliomyelitis patients share a similar hip flexion contracture due to their common ITB contracture, and surgical correction for poliomyelitis contractures was reported by Barr (2) to be 92%, and 86% by Hogshead and Ponseti (25). We have applied the same surgical principles utilized for poliomyelitis patients in our SCI cohort, and according to our reports, the index procedure was effective in controlling the hip flexion, abduction, and external rotation deformity in SCI patients as well.

The senior surgeon evolved his surgical technique because of the variability in terms of severity and complexity of the hip flexion contracture seen in each individual. Initially, an aggressive and complete release of the hip musculatures was utilized, and if residual deformity remained, an additional anterior capsulotomy was performed. However, for one patient, this provided insufficient correction intraoperatively, and therefore, by the discretion of the senior surgeon, he adopted the pelvic osteotomy used in the Campbell procedure (18, 26). This would further decompress the hip abduction muscles and provide complete hip correction in severe patients. Hip instability or displacement was skilfully neglected as femoral stabilization procedures were thought to provide little functional implications for the anesthetic SCI patient who had paralyzed hip abductors (5).

The management of lumbar hyperlordosis in the SCI population is not described in the literature. Barr (2) and Hogshead and Ponseti (25) reported LL correction for poliomyelitis patients by utilizing the historic ITB and erector spinae transfer technique. However, both studies were of ambulatory patients and lacked quantitative measurement to verify the degrees of correction. Furthermore, they did not describe any associated hip pathologies that are commonly seen in poliomyelitis patients. We observed that surgical correction of the SCI-related hip flexion contracture was associated with spontaneous resolution of their increased LL. This hip–spine relationship was first suggested by Offierski et al. (9), stating that bilateral hip flexion contractures in poliomyelitis may anteriorly angulate the pelvis and produce a compensatory increased LL to maintain an upright trunk. They further hypothesized that the correction of a fixed hip flexion deformity would correct the secondary spinal hyperlordosis. Irwin (8) additionally reported that it would be a surgical disaster to manage the lumbar spine lordosis and scoliosis in poliomyelitis patients before the hip deformity had been alleviated.

To date, the literature has not directly investigated the effects of surgical treatment in terms of the relationship between the hip and sagittal alignment of the spine in paraplegics caused by SCI as well as poliomyelitis. However, higher incidences of residual soft tissue contractures in patients with failed spinal deformity correction in SCI-related paraplegia have been briefly mentioned. Moe (27) accounted for the loss of surgical scoliosis correction to be because of the failure to initially correct all severe soft-tissue contractures of the lower extremity. Mayfield et al. (10) observed that patients managed for a lumbar hyperlordosis deformity were associated with a secondary hip flexion contracture, and we believe further consideration must be made for these contractures potentially being the primary pathology. Hwang et al. (14) reported an uncorrected LL despite spinal surgery, and in our opinion, this may possibly be due to untreated ITB contracture-related problems. Currently, the literature is too sparse to draw any significant conclusions regarding the relationship between SCI-related hip and spine deformities. Nevertheless, in our study, an association between lordosis improvements secondary to hip deformity correction was found. Therefore, we believe that careful attention should be paid for any underlying hip pathology when planning surgical options for SCI-related spinal deformities.

After discussion with a multidisciplinary team and the parents, a decision was made for the scoliosis to not be surgically managed before the hip surgery, and it was concluded that the functional deficits of the patients were primarily due to the hip flexion–abduction contracture. At the final follow-up, the Cobb angle increased by a mean of 20.1° in our series. In this study, we could not elicit a relationship between the increase in scoliotic angle and the exacerbation of the hip status of the patient. For the three patients whose hips progressed into subluxation or dislocation at the final follow-up, they interestingly saw a lesser degree of worsening of their scoliosis at the latest follow-up. Furthermore, the two patients (patients 9 and 11) who experienced aggravated scoliosis did not have any discernible hip flexion deformity or worsened hip status noted at the latest follow-up. Additionally, no relationship was found between the lordosis correction and scoliosis progression. Given the vitality of a neutral sagittal alignment in the preservation of SCI-related scoliosis correction, it seems unlikely that the improved neutralized lordosis would have a major influence on the progression of the scoliosis (28). Therefore, we believe that the SCI-related paraplegic scoliosis may be accounted for by the pathophysiology of the SCI-related neuromuscular imbalances, gravitational forces on the seated paraplegic, and physiological changes in the growing child (29, 30). Nevertheless, as our index operation does not ensure stabilization of the scoliosis itself, future spinal correction may be deemed necessary in severe cases for the overall comfort of the patients.

Unfortunately, there are currently no validated functional instruments specific for assessing the quality of life for spine and hip problems in SCI patients. We attempted to compensate for this, by utilizing subdomains of the non-specific functional measures of the SCIM, FIM, and Barthel Index to evaluate the preoperative and postoperative functional status of the patients (23, 24). Although hip deformity correction in our series provided improved physical examination findings and radiographic results, for all three functional measures, the score was not changed over the average 68-month follow-up period. However, it should be noted that all parents and patients subjectively reported increases in their function and quality of life with improvements in sitting tolerance, sleeping habits, and wheelchair function throughout the follow-up period. We believe this discrepancy may be due to the limitations of using a non-specific functional measure to accurately identify and quantify the progress for a unique cohort of paraplegic SCI patients. Until a validated and reliable functional and quality of life outcome measure is created for paraplegic SCI patients, it may be difficult to objectively review the functional outcomes of the index operation.

In addition to the above-mentioned limitation, there are several more limitations with this study. Firstly, this study has known limitations and biases relating to a retrospective design, small sample size, and product of the unique diagnosis. Secondly, consistent serial follow-up radiographs were unavailable for all patients. Final follow-up radiographs were used for lordosis correction, and the increase in LL associated with skeletal maturity may mask the improvement seen after the index surgery (31–33). Thirdly, additional variables that may exacerbate patient function such as pelvic obliquity and pathological fractures were not investigated in this study, and future studies exploring the effects of our procedure and influence of these factors in SCI patients will be necessary. Fourthly, limitations of a retrospective study design allowed for varying follow-up times between the patients, and this variation may affect the outcomes of the deformity correction.

For paraplegic SCI patients with debilitating hip flexion contractures, corrective hip surgery was beneficial by providing an increased hip range of motion and enabling comfortable sitting and lying positions. We found an association between treating the severe hip flexion–abduction–external rotation contracture and secondary correction of the lumbar hyperlordosis of a patient. We recommend the spine surgeon carefully consider the hip pathology when managing SCI-associated lumbar hyperlordosis.

## Data Availability Statement

The original contributions presented in the study are included in the article/supplementary material, further inquiries can be directed to the corresponding author/s.

## Ethics Statement

The studies involving human participants were reviewed and approved by Severance Hospital Institutional Review Board. Written informed consent from the participants' legal guardian/next of kin was not required to participate in this study in accordance with the national legislation and the institutional requirements.

## Author Contributions

HK: conception and design, supervision, and project administration. HK and K-BP: technical and material support. WD and BP: data curation. IR and WD: formal analysis and interpretation of data. IR: drafting of the article. HK, IR, and K-BP: critical revision and editing. All authors contributed to the article and approved the submitted version.

## Conflict of Interest

The authors declare that the research was conducted in the absence of any commercial or financial relationships that could be construed as a potential conflict of interest.
